# Supplementary material for: Three-Dimensional Flower-like MoS2 Nanosheets Grown on Graphite as High-Performance Anode Materials for Fast-Charging Lithium-Ion Batteries
Source: Materials (Basel). 2023 May 27;16(11):4016. doi: 10.3390/ma16114016 (PMC10254478; doi:10.3390/ma16114016)
Supplement: Supplementary file 1 [file materials-16-04016-s001.zip › materials-2383181-supplementary.pdf]

Supplementary materials

Three-Dimensional Flower-like MoS_2 Nanosheets Grown on Graphite as High-Performance Anode Materials for Fast-Charging Lithium-ion Batteries

Yeong A. Lee,[1,2,†] Kyu Yeon Jang,[1,3,†] Jaeseop Yoo,[2,†] Kanghoon Yim,[4] Wonzee Jung,[4,5] Kyu-Nam Jung,[1] Chung-Yul Yoo,[6] Younghyun Cho,[7] Jinhong Lee,[1] Myung Hyun Ryu,[1] Hyeyoung Shin,[2,*] Kyubock Lee,[2,*] Hana Yoon,[1,*]

[1] *Korea Institute of Energy Research (KIER), Daejeon 34129, Republic of Korea*

[2] *Graduate School of Energy Science and Technology (GEST), Chungnam National University, Daejeon 34134, Republic of Korea*

[3] *Department of Advanced Energy Technologies and System Engineering, Korea University of Science and Technology (UST), Daejeon 34113, Republic of Korea*

[4] *Computational Science and Engineering Laboratory, Korea Institute of Energy Research (KIER), Daejeon 34129, Republic of Korea*

[5] *Department of Physics, Chungnam National University, 99 Daehak-ro, Daejeon 34134, Republic of Korea*

[6] *Department of Chemistry, Mokpo National University, Muan-gun 58554, Republic of Korea*

[7] *Department of Energy Systems, Soonchunhyang University, Asan 31538, Republic of Korea*

[†] These authors contributed equally to this work.

* Corresponding authors.

Email addresses: shinhy@cnu.ac.kr (H. Shin), kyubock.lee@cnu.ac.kr (K. Lee), hanayoon@kier.re.kr (H. Yoon)

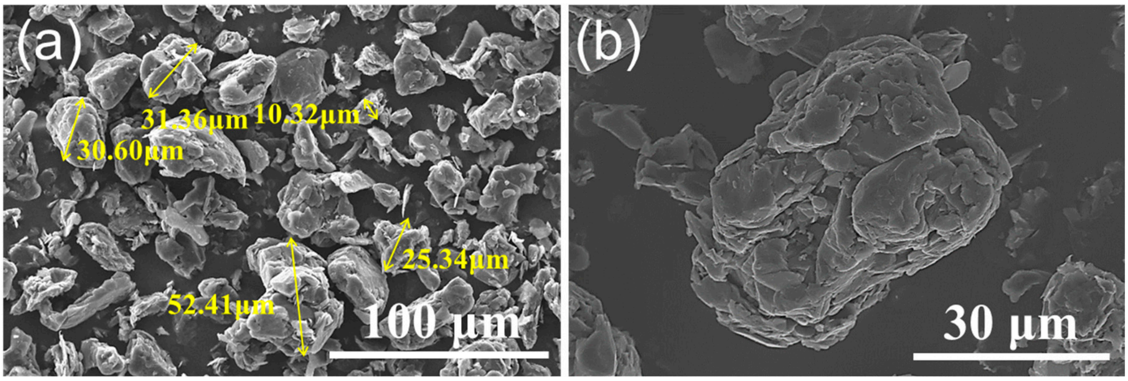

Figure S1. SEM images of the pristine artificial graphite (AG) obtained at (a) low magnification (scale bar: 100 μm) and (b) enlarged magnification (scale bar: 30 μm).

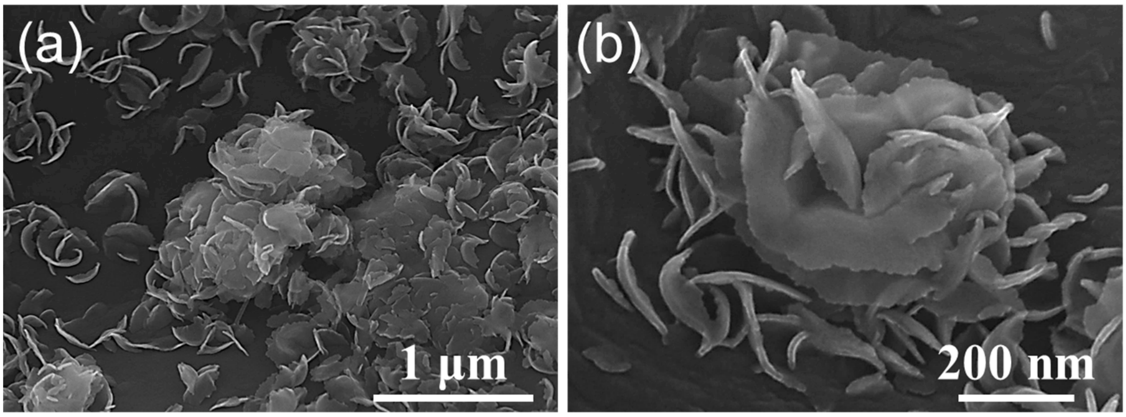

Figure S2. SEM images of the MoS_2@AG composite obtained at various enlarged magnifications.

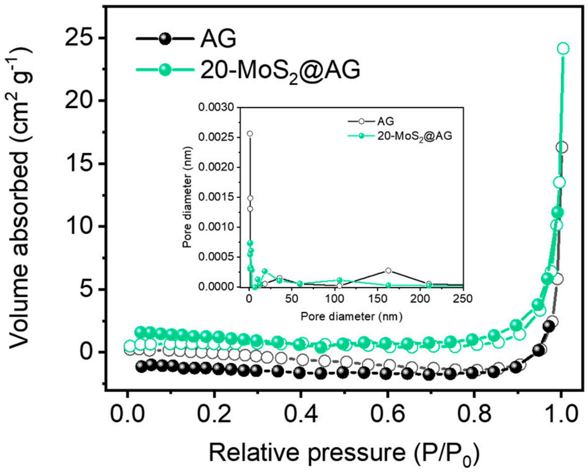

	Specific surface area (m^2/g)	Mean pore diameter (nm)	Total pore volume (cm^3/g)
AG	1.15	29.28	0.008
MoS_2/AG	2.98	21.48	0.016

Figure S3. N_2 adsorption and desorption isotherms of pristine graphite (AG) and 20−MoS_2@AG composite. The inset shows their pore size distribution curves.

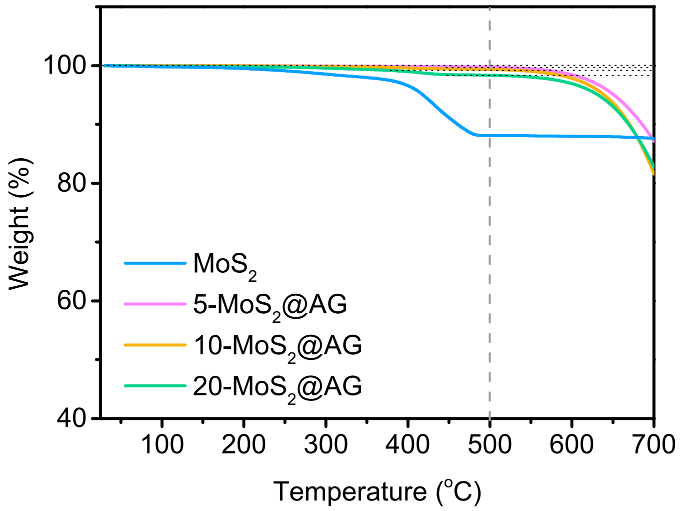

Figure S4. TGA curves of pristine MoS_2, 5−MoS_2@AG, 10−MoS_2@AG, and 20−MoS_2@AG composites with a heating rate of 10 °C min^{-1} in air.

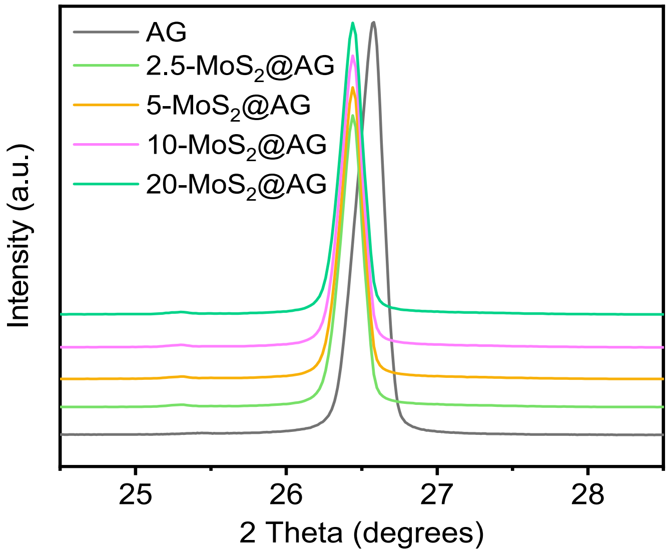

Figure S5. XRD patterns of MoS_2@AG composites.

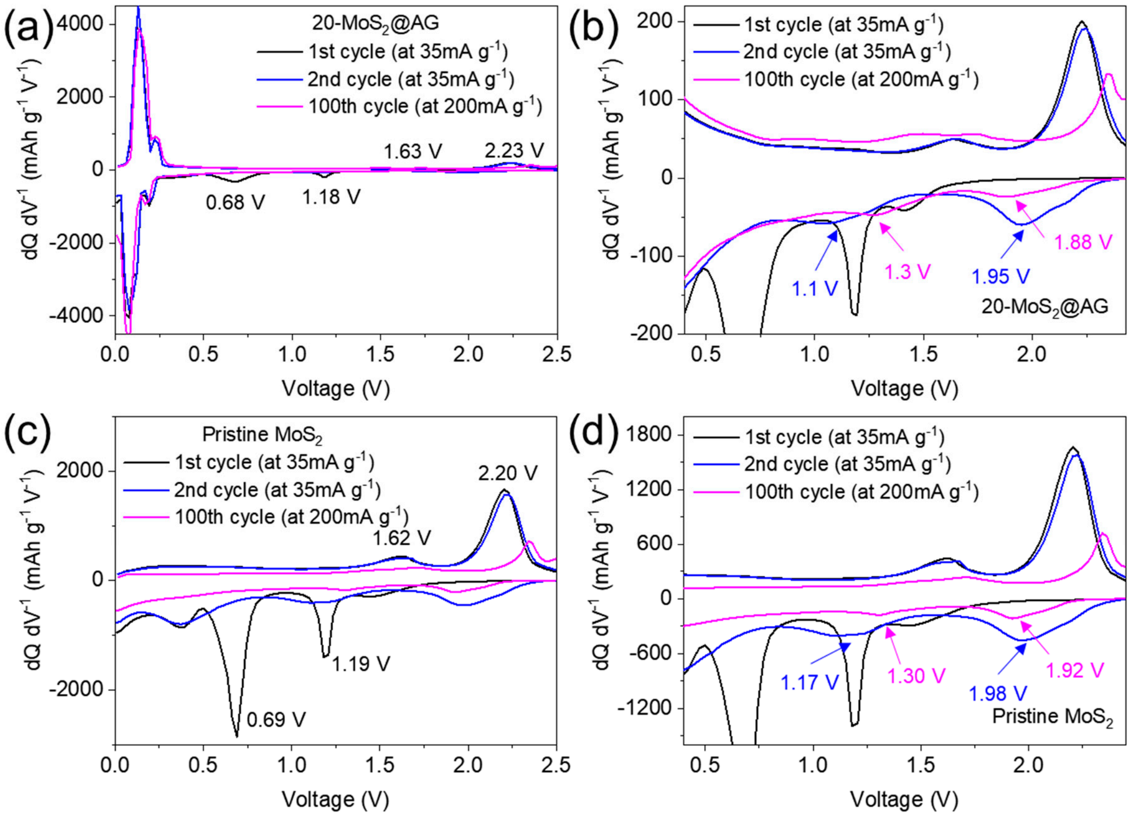

Figure S6. The differential voltage profiles of (a,b) the 20−MoS_2@AG electrode and (c,d) pristine MoS_2.

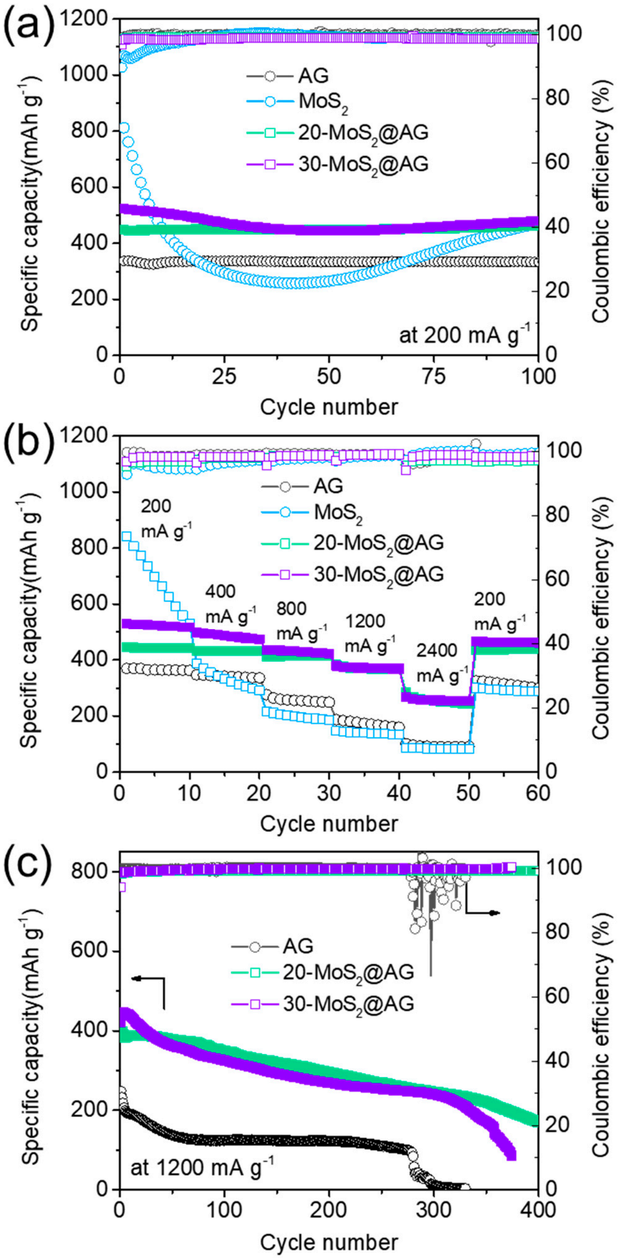

Figure S7. (a) Cycle performance at a current density of 200 mA g^{-1}, (b) Rate performance comparison of pristine AG, pristine MoS_2, 20−MoS_2@AG, and 30−MoS_2@AG electrodes at different current densities ranging from 200 to 2400 mA g^{-1}, and (c) Cycle performance at a high current density of 1200 mA g^{-1} for pristine AG, 20−MoS_2@AG, and 30−MoS_2@AG electrodes.

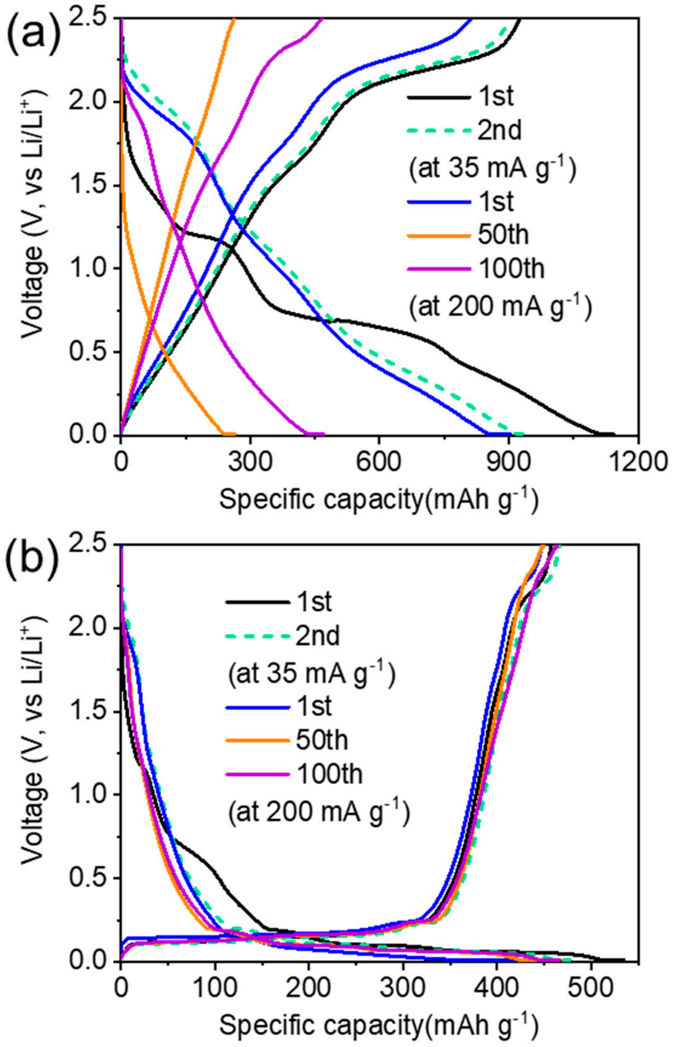

Figure S8. Galvanostatic charge and discharge curves of (a) pristine MoS_2 and (b) 20−MoS_2@AG electrodes.

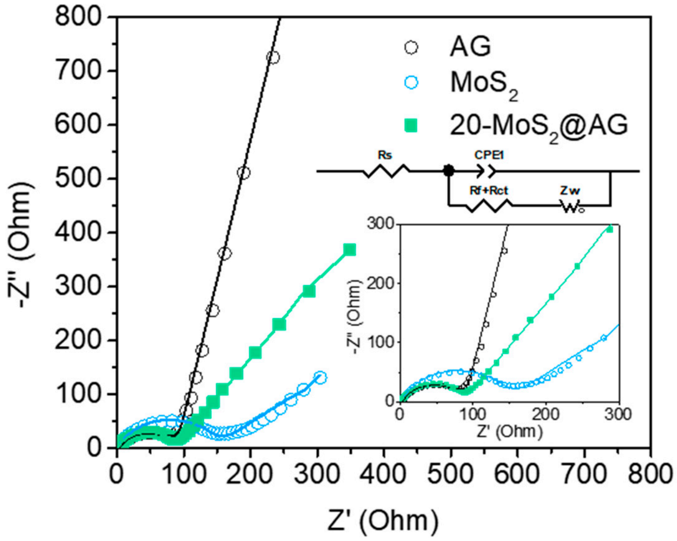

Figure S9. Nyquist plots from EIS data (symbols) and fitting results (solid lines) of pristine AG, pristine MoS_2, and 20–MoS_2@AG obtained after 2 cycles at a current density of 35 mA g^{-1}.

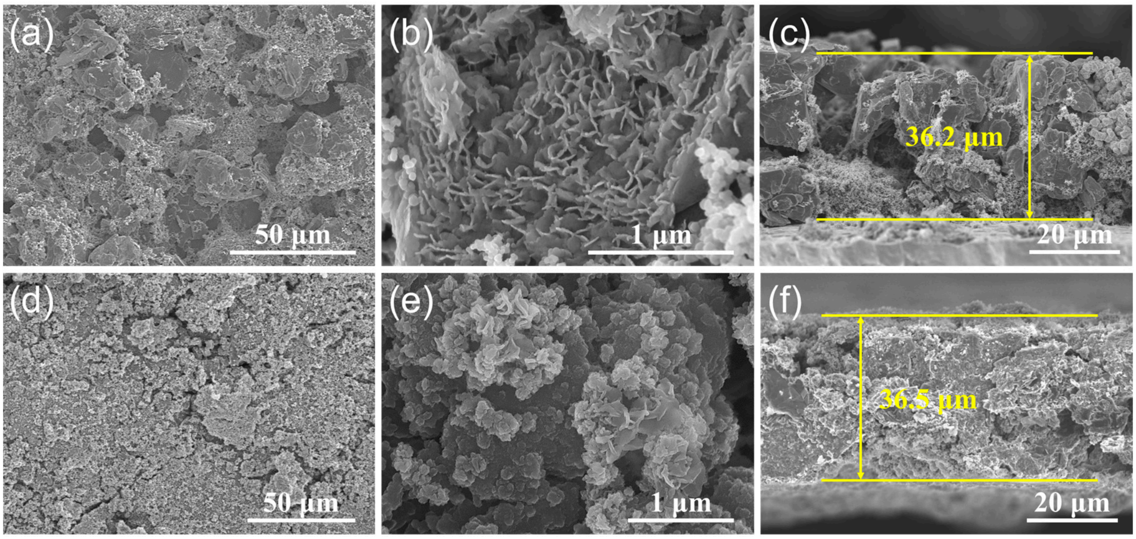

Figure S10. Ex situ SEM images of the 20−MoS_2@AG electrodes before and after 300 cycles at a current density of 200 mA g^{-1}; (a-c) before cycling and (d-f) after cycling. Panels (c) and (f) of Figure S10 show cross-sectional SEM images of the electrode before and after cycling, respectively.

Table S1. Comparison of the initial capacities and rate performance for pristine graphite, pristine MoS_2, 5−MoS_2@AG, 10−MoS_2@AG, 20−MoS_2@AG, and 30−MoS_2@AG composites.

Materials	Initial capacity / Coulombic efficiency	MoS_2 contribution	Reversible capacity	Capacity retention of initial value at 200 mA g^{-1} $\left(\frac{Capacity_{@1200mA/g}}{Capacity_{@200mA/g}} \times 100\right)$
	(@ 35 mA g^{-1})	(@ 35 mA g^{-1})	(@ 200 mA g^{-1})	(@ 1200 mA g^{-1})
**Pristine graphite**	373.3 mAh g^{-1} (91.2 %)	-	365.5 mAh g^{-1}	47.2 % (172.6 mAh g^{-1})
**Pristine MoS_2**	921.3 mAh g^{-1} (81.4%)	-	699.5 mAh g^{-1}	20.0 % (139.7 mAh g^{-1})
**30-MoS_2@AG**	536.8 mAh g^{-1} (84.0%)	163.5 mAh g^{-1}	522.8 mAh g^{-1}	71.0 % (371.4 mAh g^{-1})
**20-MoS_2@AG**	494.1 mAh g^{-1} (86.1%)	121 mAh g^{-1}	441.7 mAh g^{-1}	84.0 % (371.0 mAh g^{-1})
**10-MoS_2@AG**	460.7 mAh g^{-1} (85.7 %)	87 mAh g^{-1}	426.1 mAh g^{-1}	84.7 % (361.1 mAh g^{-1})
**5-MoS_2@AG**	414.6 mAh g^{-1} (86.0%)	41 mAh g^{-1}	401.8 mAh g^{-1}	88.7 % (356.4 mAh g^{-1})

Table S2. Comparison of LIB electrochemical performance for transition metal sulfides (MS_x) and transition metal oxides (MO_x)-based composites.

Materials	MS_x or MO_x content (wt%)	Specific capacity (mAh g^{-1}) *(@ Current density (mA g^{-1}))*	Ref
**20-MoS_2@AG**	~ 5	463.2 (@*200*, at 1st cycle)	This work
		400.0 (@*1200*, at 1st cycle)	
**TiO_{2-x}/Graphite**	8	316.1 (@*70*, at 1st cycle)	[12]
		290.4 *(@1750,* at 1st cycle)	
**c-MoO_2/Natural graphite**	2.2	365.6 *(@70,* at 1st cycle)	[14]
**c-Fe_3O_4/Natural graphite**	2.9	356.4 *(@70*, at 1st cycle)	
**Spherical graphite/Fe_2O_3**	8.7	455 *(@100*, at 1st cycle)	[15]
**MoS_2/C**	77	753 *(@ 100*, at 1st cycle)	[21]
**MoS_2/Exfoliated graphene**	95.3	1543 *(@ 50*, at 1st cycle)	[22]
		1250 *(@ 1000*, at 150th cycle)	
**MoS_2@Carbon nanofibers**	54.857	938.8 *(@200*, at 1st cycle)	[38]
**MoS_2@C/MoS_2**	57.3	802 *(@ 100*, at 1st cycle)	[39]
**$MoSe_2$/Graphite**	70	671 *(@ 100*, at 1st cycle)	[40]
**MoS_2/Graphene**	70	807 *(@ 100,* at 1st cycle)	[41]
**MoS_2/Graphite**		559.3 *(@ 100,* at 1st cycle)	
**MoS_2/Graphene**	67.3	662 *(@200*, at 3rd cycle)	[42]

Table S3. EIS fitting parameters for AG, MoS_2, and 20−MoS_2@AG before the cycling tests.

	**AG**	**MoS_2**	**20−MoS_2@AG**
**R_S (Ω)**	3.04 (±0.05)	1.39 (±0.06)	1.33 (±0.01)
**CPE1 ($\Omega^{-1}\cdot s^{-n}$)**	2.37×10^{-5} ($\pm 1.5 \times 10^{-6}$)	2.27×10^{-5} ($\pm 1.8 \times 10^{-6}$)	1.17×10^{-5} ($\pm 3.4 \times 10^{-7}$)
**n1**	0.79 (±0.01)	0.77 (±0.01)	0.86 (±0.01)
**$R_f + R_{ct}$ (Ω)**	101 (±0.2)	128 (±2.6)	128 (±1.4)
**Z_wR (Ω)**	48.7 (±8.2)	110 (±10)	51.8 (±6.0)
**Z_wT (s)**	0.036 ($\pm 1.0 \times 10^{-3}$)	0.195 ($\pm 1.0 \times 10^{-3}$)	0.054 ($\pm 1.0 \times 10^{-3}$)
**Z_wP**	0.48 (±0.01)	0.44 (±0.01)	0.46 (±0.01)

Table S4. EIS fitting parameters for AG, MoS_2, and 20−MoS_2@AG after 2 cycles.

	**AG**	**MoS_2**	**20−MoS_2@AG**
**R_S (Ω)**	3.29 (±0.03)	1.46 (±0.03)	1.40 (±0.03)
**CPE1 ($\Omega^{-1}\cdot s^{-n}$)**	3.25×10^{-5} ($\pm 1.50 \times 10^{-6}$)	1.54×10^{-5} ($\pm 6.9 \times 10^{-7}$)	2.61×10^{-5} ($\pm 1.59 \times 10^{-6}$)
**n1**	0.75 (±0.01)	0.79 (±0.01)	0.77 (±0.01)
**$R_f + R_{ct}$ (Ω)**	76.3 (±1.3)	139 (±4)	76.8 (±2.3)
**Z_wR (Ω)**	42.4 (±4.5)	192.6 (±35.6)	35.6 (±10.1)
**Z_wT (s)**	0.026 ($\pm 1.0 \times 10^{-3}$)	3.93 (±1.88)	0.06 (±0.03)
**Z_wP**	0.44 (±0.01)	0.25 (±0.02)	0.31 (±0.01)
